# Editorial to the Special Issue “Human Bodywork: Applications in Health, Disease, and Rehabilitation”

**DOI:** 10.3390/biology12030451

**Published:** 2023-03-15

**Authors:** Redha Taiar

**Affiliations:** MATériaux et Ingénierie Mécanique (MATIM), Université de Reims Champagne Ardenne, F-51100 Reims, France; redha.taiar@univ-reims.fr

## 1. Introduction

In this research topic, the question concerning how the human body functions through the musculoskeletal system was addressed. The management of human movements requires the control of different body segments which can be coordinated in multiple ways to perform a defined task. The way that the biological system functions in relation to human movement is particularly complex. Therefore, this requires a deep understanding of the interactions between physical sciences (metrology), sciences and technologies of information, and sciences of life (materials, fabrics, organs, and members). The purpose of this Special Issue (SI) is to specify the principles of physiology, functional anatomy, and mechanics to explore and understand biological problems. This can be augmented by human research that facilitates the optimization of human behavior. The researchers’ objective for this SI was to summarize the most important parameters influencing human performance, in relation to health sciences in the lives of people from all age groups. In this Special Issue, the researchers were inspired by journal papers that aimed to promote the latest research in the fields of health, quality of life improvement, and sport rehabilitation, and to highlight the recent recommendations. This will help to prevent functional decline and frailty throughout the course of life. This prevention is in the form of a perspective approach via the utilization of the latest research applied in general health and it targets all stages of life. In addition, contributions to this SI aimed at prevention, improved performance, the management of diseases, modelization, simulation, quantification, and the computation of the musculoskeletal system, thus allowing researchers to quantify and improve the disparate parameters characterizing movement in different cases such as sport level, work, and patients’ daily lives. The aim of this SI is to effectively combine and coordinate research and results in order to understand and improve human bodywork in medicine, in sport, and at work.

In this Special Issue, thirty-one articles were included which addressed those questions. Twenty-six of them are original trials, along with one review, one systematic review, one protocol, and one tutorial. Herein, we summarize their major contributions according to the subject categories. Overall, the studies showed the importance of this subject in improving the quality of life of patients’ lives by expanding upon current evidence; moreover, the studies indicated directions for future research.

The articles all focus on the optimization of the musculoskeletal system and the improvement of patients’ quality of life. Many fascinating, high-quality methodologies have been developed across the different articles. We shall now summarize their major contributions according to the subject categories.

In the first category, Freehill et al. [1] and Servant et al. [2] focused on surgery of the musculoskeletal system. In the second category, several authors focused on rehabilitation programs and the improvement of body movement. From the contributing studies in this category, we can mention Martins et al. [3], Cuenca-Zaldivar et al. [4]. Dos Santos et al. [5], Maciejczyk et al. [6], Dufournet et al. [7], Shabat et al. [8], An et al. [9], Martínez-Jiménez et al. [10], Wang et al. [11], Ofran et al. [12], Wang et al. [13], Rostron et al. [14], Silva Caiado et al. [15], Arenales Arauz et al. [16], Souto Braz et al. [17], and Van Heuvelen et al. [18] with an Reporting Guidelines for Whole-Body Vibration Studies in Humans, Animals and Cell Cultures. In the third category, the optimization of athlete performance was discussed in the papers of Brambilla et al. [19], Strutzenberger et al. [20], and Kiers et al. [21]. In the fourth category, the authors focused on the modelization of the musculoskeletal system by Computational Analysis, the Finite Element Method, and the application of a Machine Learning Algorithms approach on rehabilitation, with submissions by Hong et al. [22], Wang et al. [23], Zhang et al. [24], Antunes et al. [25], Da Cunha de Sá-Caputo et al. [26], Cheung et al. [27], and Lu et al. [28]. In the fifth category, two studies, namely, Rapin et al. [29] and Davahli et al. [30], focused on the impact of COVID-19 on patients in France, USA, and Japan, and one author, De-Yñigo-Mojado et al. [31], focused on the effects of facial skin hair on the fit factor of surgical masks.

## 2. Conclusions

This Special Issue is highly informative as it relates to the latest research in the field of «health disease and rehabilitation»; moreover, it allows readers to deepen their knowledge and understanding of the complexity of the musculoskeletal system’s mechanism.
